# Crystal structure of 2-{[2-(3-phenyl­allyl­idene)hydrazin-1-yl]thio­carbonyl­sulfanylmeth­yl}pyridinium chloride

**DOI:** 10.1107/S1600536814023228

**Published:** 2014-10-29

**Authors:** May Lee Low, Thahira Begum S. A. Ravoof, Mohamed Ibrahim Mohamed Tahir, Karen A. Crouse, Edward R. T. Tiekink

**Affiliations:** a Department of Chemistry, Universiti Putra Malaysia, 43400 Serdang, Malaysia; b Department of Chemistry, Cape Breton University, Sydney, Nova Scotia, B1P 6L2, Canada; c Department of Chemistry, University of Malaya, 50603 Kuala Lumpur, Malaysia

**Keywords:** crystal structure, hydrogen bonding, *S*-substituted di­thio­carbaza­tes, salt

## Abstract

In the title salt of an *S*-substituted di­thio­carbazate, C_16_H_16_N_3_S_2_^+^·Cl^−^, the dihedral angles between the almost planar (r.m.s deviation = 0.005 Å) central CN_2_S_2_ residue and the terminal pyridinium and phenyl rings are 80.09 (11) and 3.82 (11)°, respectively, indicating the cation has an L-shape; the amine H and thione S atoms are *syn*. The conformation about each of the imine [1.376 (3) Å] and ethene [1.333 (4) Å] bonds is *E*. The shortened C—C bond [1.444 (4) Å] linking the double bonds is consistent with conjugation in this part of the mol­ecule. In the crystal, supra­molecular layers with a jagged topology are formed by charged-assisted amine-H⋯Cl^−^ and pyridinium-N^+^—H⋯Cl^−^ hydrogen bonds. The layers stack along the *a* axis with no specific directional inter­actions between them.

## Related literature   

For general background to related Schiff bases formed between *S*-substituted di­thio­carbaza­tes and cinnamaldehyde, see: Low *et al.* (2013 ▶). For the biological activity of similar sulfur/nitro­gen-containing Schiff base derivatives, see: Khoo *et al.* (2014 ▶). For the synthetic procedure, see: Crouse *et al.* (2004 ▶).
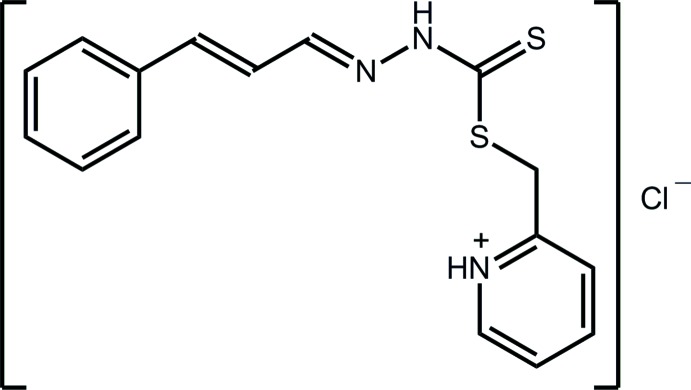


## Experimental   

### Crystal data  

C_16_H_16_N_3_S_2_^+^·Cl^−^
*M* *_r_* = 349.89
Orthorhombic, 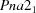

*a* = 24.2206 (8) Å
*b* = 8.2838 (2) Å
*c* = 8.3206 (4) Å
*V* = 1669.43 (11) Å^3^
*Z* = 4
Cu *K*α radiation
μ = 4.35 mm^−1^
*T* = 150 K
0.12 × 0.05 × 0.01 mm

### Data collection  

Agilent Xcaliber Eos Gemini diffractometer
Absorption correction: multi-scan (*CrysAlis PRO*; Agilent, 2011 ▶) *T*_min_ = 0.80, *T*_max_ = 0.96
5463 measured reflections
2460 independent reflections
2327 reflections with *I* > 2σ(*I*)
*R*_int_ = 0.022

### Refinement  

*R*[*F* ^2^ > 2σ(*F* ^2^)] = 0.031
*wR*(*F* ^2^) = 0.078
*S* = 1.04
2460 reflections
205 parameters
3 restraints
H atoms treated by a mixture of independent and constrained refinement
Δρ_max_ = 0.37 e Å^−3^
Δρ_min_ = −0.23 e Å^−3^
Absolute structure: Flack (1983 ▶), 971 Friedel pairs
Absolute structure parameter: −0.009 (16)

### 

Data collection: *CrysAlis PRO* (Agilent, 2011 ▶); cell refinement: *CrysAlis PRO*; data reduction: *CrysAlis PRO*; program(s) used to solve structure: *SHELXS97* (Sheldrick, 2008 ▶); program(s) used to refine structure: *SHELXL97* (Sheldrick, 2008 ▶); molecular graphics: *ORTEP-3 for Windows* (Farrugia, 2012 ▶) and *DIAMOND* (Brandenburg, 2006 ▶); software used to prepare material for publication: *publCIF* (Westrip, 2010 ▶).

## Supplementary Material

Crystal structure: contains datablock(s) I. DOI: 10.1107/S1600536814023228/hb7295sup1.cif

Structure factors: contains datablock(s) I. DOI: 10.1107/S1600536814023228/hb7295Isup2.hkl

Supporting information file. DOI: 10.1107/S1600536814023228/hb7295Isup3.cml

**Fig. 1.**
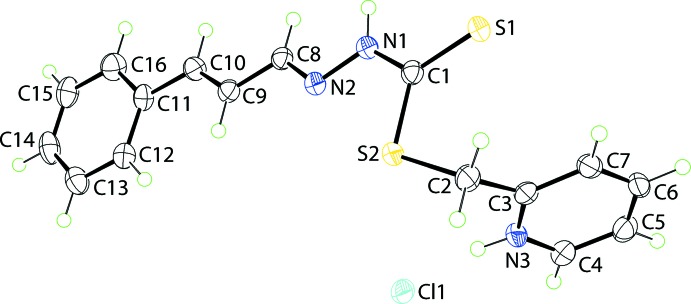
The molecular structure of the title compound showing displacement ellipsoids at the 50% probability level.

**Fig. 2.**
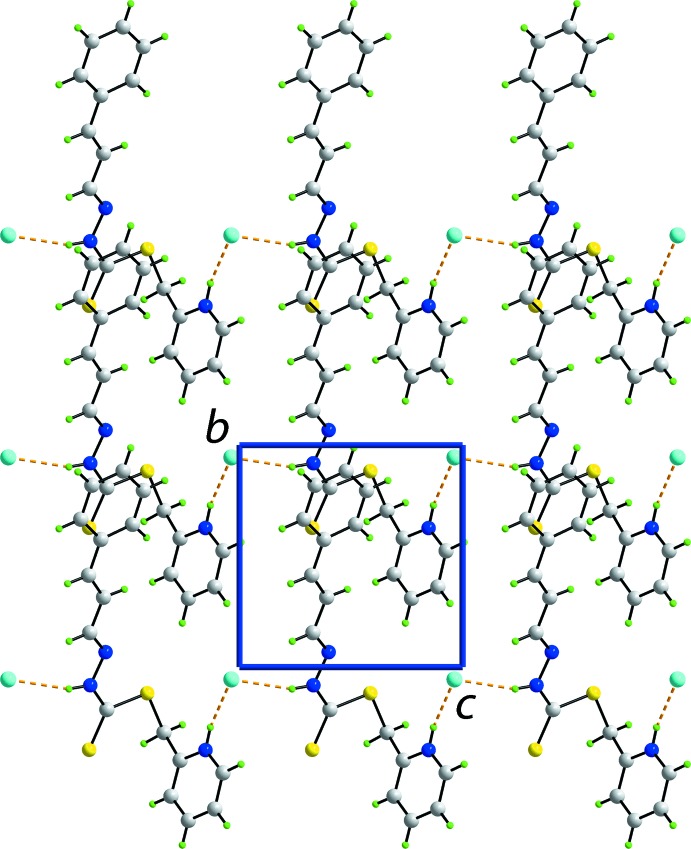
A view of the supramolecular layer in the bc plane mediated by charge-assisted N—H···Cl hydrogen bonds (orange dashed lines).

. DOI: 10.1107/S1600536814023228/hb7295fig1.tif
The mol­ecular structure of the title compound showing displacement ellipsoids at the 50% probability level.

bc . DOI: 10.1107/S1600536814023228/hb7295fig2.tif
A view of the supra­molecular layer in the *bc* plane mediated by charge-assisted N—H⋯Cl hydrogen bonds (orange dashed lines).

CCDC reference: 1030368

Additional supporting information:  crystallographic information; 3D view; checkCIF report

## Figures and Tables

**Table 1:** Hydrogen-bond geometry (, )

*D*H*A*	*D*H	H*A*	*D* *A*	*D*H*A*
N1H1*N*Cl1^i^	0.88(2)	2.29(2)	3.104(3)	153(3)
N3H3*N*Cl1	0.88(2)	2.13(2)	2.9833(19)	163(3)

Symmetry code: (i) 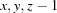.

## supplementary crystallographic information

### S5. Synthesis and crystallization

An equimolar amount of *trans*-cinnamaldehyde (1.26 ml) was added to a solution of *S*-2-picolyldi­thio­carbazate hydro­chloride (2.36 g, 0.01 mol), prepared by literature methods (Crouse *et al.*, 2004), dissolved in hot absolute ethanol (100 ml). The mixture was heated while being stirred to reduce it to half the original volume and then cooled. The yellow compound was filtered, washed with absolute ethanol then dried over silica gel. Single crystals of diffraction quality were obtained after recrystallisation from its methano­lic solution. % yield = 90%. *M*.pt = 465-466 K. HR—MS: *m*/*z* = [M—Cl+H]^+^ Calcd. 314.07802, Found 314.07826. RP-HPLC retention time, RT = 15 min. FT—IR: ν (cm^–1^) = 3048 (w), 1621 (m), 1033 (m), 978 (s) and 951 (m). UV-Vis in DMSO: λ_max_ nm (log ε) = 360 (4.65), 377 (4.52, sh), 344 (4.53, sh). ^1^H NMR (300 MHz, DMSO-d_6_) δ = 13.53 (*s*, 1H), 8.78 (*d*, J = 5.2, 1H), 8.37 (*t*, J = 7.8, 1H), 8.14 (*d*, J = 9.4, 1H), 7.98 (*d*, J = 8.0, 1H), 7.86 - 7.78 (*m*, 1H), 7.66 (*d*, J = 6.7, 2H), 7.38 (*q*, J = 6.3, 3H), 7.21 (*d*, J = 16.0, 1H), 6.97 (*dd*, J = 16.0, 9.4, 1H), 4.87 (*s*, 2H); the N-bound H was not observed. ^13^C NMR (75 MHz, DMSO-d_6_) δ = 193.66, 153.87, 150.12, 144.22, 142.97, 142.77, 135.54, 129.49, 128.90, 127.53, 126.39, 124.86, 124.04, 34.92. GC—MS (fragmentation pattern) *m*/*z* = 313, 187,130, 125, 103, 92, 77, 65, 51.

### S6. Refinement

Carbon-bound H-atoms were placed in calculated positions (C—H = 0.95 to 0.99 Å) and were included in the refinement in the riding model approximation with *U_iso_*(H) = 1.2*U_eq_*(C). The N—H H atoms were refined with N—H = 0.88±0.01 Å, and with *U_iso_*(H) = 1.2*U_eq_*(N).

### Crystal data

**Table:**

C_16_H_16_N_3_S_2_^+^·Cl^−^	*F*(000) = 728
*M**_r_* = 349.89	*D*_x_ = 1.392 Mg m^−^^3^
Orthorhombic, *P**n**a*2_1_	Cu *K*α radiation, λ = 1.54180 Å
Hall symbol: P 2c -2n	Cell parameters from 3280 reflections
*a* = 24.2206 (8) Å	θ = 4–71°
*b* = 8.2838 (2) Å	µ = 4.35 mm^−^^1^
*c* = 8.3206 (4) Å	*T* = 150 K
*V* = 1669.43 (11) Å^3^	Plate, yellow
*Z* = 4	0.12 × 0.05 × 0.01 mm

### Data collection

**Table:**

Agilent Xcaliber Eos Gemini diffractometer	2460 independent reflections
Radiation source: fine-focus sealed tube	2327 reflections with *I* > 2σ(*I*)
Graphite monochromator	*R*_int_ = 0.022
Detector resolution: 16.1952 pixels mm^-1^	θ_max_ = 67.7°, θ_min_ = 3.7°
ω scans	*h* = −29→28
Absorption correction: multi-scan (*CrysAlis PRO*; Agilent, 2011)	*k* = −7→9
*T*_min_ = 0.80, *T*_max_ = 0.96	*l* = −8→8
5463 measured reflections	

### Refinement

**Table:**

Refinement on *F*^2^	Secondary atom site location: difference Fourier map
Least-squares matrix: full	Hydrogen site location: inferred from neighbouring sites
*R*[*F*^2^ > 2σ(*F*^2^)] = 0.031	H atoms treated by a mixture of independent and constrained refinement
*wR*(*F*^2^) = 0.078	*w* = 1/[σ^2^(*F*_o_^2^) + (0.0531*P*)^2^ + 0.1085*P*] where *P* = (*F*_o_^2^ + 2*F*_c_^2^)/3
*S* = 1.04	(Δ/σ)_max_ = 0.001
2460 reflections	Δρ_max_ = 0.37 e Å^−^^3^
205 parameters	Δρ_min_ = −0.23 e Å^−^^3^
3 restraints	Absolute structure: Flack (1983), 971 Friedel pairs
Primary atom site location: structure-invariant direct methods	Absolute structure parameter: −0.009 (16)

### Special details

**Table:**

Geometry. All s.u.'s (except the s.u. in the dihedral angle between two l.s. planes) are estimated using the full covariance matrix. The cell s.u.'s are taken into account individually in the estimation of s.u.'s in distances, angles and torsion angles; correlations between s.u.'s in cell parameters are only used when they are defined by crystal symmetry. An approximate (isotropic) treatment of cell s.u.'s is used for estimating s.u.'s involving l.s. planes.
Refinement. Refinement of *F*^2^ against ALL reflections. The weighted *R*-factor *wR* and goodness of fit *S* are based on *F*^2^, conventional *R*-factors *R* are based on *F*, with *F* set to zero for negative *F*^2^. The threshold expression of *F*^2^ > 2σ(*F*^2^) is used only for calculating *R*-factors(gt) *etc*. and is not relevant to the choice of reflections for refinement. *R*-factors based on *F*^2^ are statistically about twice as large as those based on *F*, and *R*- factors based on ALL data will be even larger.

### Fractional atomic coordinates and isotropic or equivalent isotropic displacement parameters (Å^2^)

**Table:**

	*x*	*y*	*z*	*U*_iso_*/*U*_eq_	
Cl1	0.63222 (3)	−0.05537 (7)	0.96332 (11)	0.03476 (18)	
S1	0.69437 (3)	−0.37225 (7)	0.32589 (10)	0.02657 (17)	
S2	0.71117 (3)	−0.11756 (6)	0.58644 (10)	0.02635 (17)	
N1	0.65178 (9)	−0.0817 (2)	0.3311 (4)	0.0252 (5)	
H1N	0.6375 (13)	−0.098 (4)	0.235 (2)	0.030*	
N2	0.64340 (9)	0.0675 (2)	0.3995 (3)	0.0259 (5)	
N3	0.67390 (9)	−0.3736 (2)	0.8473 (3)	0.0268 (5)	
H3N	0.6674 (12)	−0.2714 (16)	0.869 (4)	0.032*	
C1	0.68354 (9)	−0.1909 (3)	0.4053 (4)	0.0238 (6)	
C2	0.74713 (11)	−0.2944 (3)	0.6613 (4)	0.0297 (7)	
H2A	0.7679	−0.3442	0.5717	0.036*	
H2B	0.7742	−0.2598	0.7434	0.036*	
C3	0.70984 (10)	−0.4195 (3)	0.7339 (4)	0.0264 (6)	
C4	0.64075 (12)	−0.4762 (3)	0.9260 (4)	0.0306 (7)	
H4	0.6164	−0.4375	1.0068	0.037*	
C5	0.64217 (12)	−0.6385 (3)	0.8889 (4)	0.0339 (7)	
H5	0.6189	−0.7130	0.9432	0.041*	
C6	0.67820 (12)	−0.6902 (3)	0.7709 (4)	0.0327 (7)	
H6	0.6798	−0.8014	0.7435	0.039*	
C7	0.71189 (12)	−0.5813 (3)	0.6926 (4)	0.0301 (7)	
H7	0.7364	−0.6171	0.6108	0.036*	
C8	0.60714 (9)	0.1556 (3)	0.3289 (4)	0.0241 (6)	
H8	0.5884	0.1164	0.2365	0.029*	
C9	0.59502 (10)	0.3146 (3)	0.3905 (4)	0.0257 (6)	
H9	0.6141	0.3502	0.4837	0.031*	
C10	0.55839 (10)	0.4140 (3)	0.3235 (4)	0.0272 (6)	
H10	0.5403	0.3757	0.2297	0.033*	
C11	0.54303 (10)	0.5752 (3)	0.3785 (4)	0.0276 (7)	
C12	0.56395 (11)	0.6430 (3)	0.5201 (4)	0.0287 (7)	
H12	0.5893	0.5831	0.5834	0.034*	
C13	0.54828 (11)	0.7955 (3)	0.5692 (5)	0.0346 (7)	
H13	0.5632	0.8404	0.6648	0.042*	
C14	0.51043 (12)	0.8835 (3)	0.4779 (5)	0.0381 (8)	
H14	0.4990	0.9877	0.5123	0.046*	
C15	0.48967 (11)	0.8192 (3)	0.3381 (5)	0.0376 (8)	
H15	0.4641	0.8793	0.2755	0.045*	
C16	0.50597 (11)	0.6662 (4)	0.2882 (5)	0.0365 (8)	
H16	0.4916	0.6231	0.1910	0.044*	

### Atomic displacement parameters (Å^2^)

**Table:**

	*U*^11^	*U*^22^	*U*^33^	*U*^12^	*U*^13^	*U*^23^
Cl1	0.0546 (4)	0.0238 (3)	0.0258 (4)	0.0111 (3)	0.0018 (4)	0.0001 (3)
S1	0.0320 (3)	0.0197 (3)	0.0279 (4)	0.0013 (2)	0.0003 (3)	−0.0026 (3)
S2	0.0362 (3)	0.0192 (3)	0.0237 (4)	0.0015 (2)	−0.0028 (3)	−0.0013 (3)
N1	0.0312 (10)	0.0209 (10)	0.0235 (15)	0.0022 (8)	0.0006 (12)	−0.0007 (10)
N2	0.0335 (11)	0.0199 (10)	0.0244 (15)	0.0009 (8)	0.0008 (11)	−0.0005 (9)
N3	0.0347 (10)	0.0189 (9)	0.0268 (16)	0.0051 (8)	−0.0042 (12)	−0.0023 (10)
C1	0.0244 (11)	0.0221 (11)	0.0250 (18)	−0.0015 (9)	0.0025 (12)	0.0017 (11)
C2	0.0318 (13)	0.0256 (13)	0.0316 (19)	0.0058 (10)	−0.0097 (14)	0.0018 (13)
C3	0.0304 (13)	0.0244 (12)	0.0244 (19)	0.0081 (10)	−0.0126 (13)	0.0018 (11)
C4	0.0357 (13)	0.0283 (12)	0.028 (2)	0.0021 (10)	−0.0020 (14)	0.0028 (12)
C5	0.0392 (14)	0.0296 (13)	0.033 (2)	−0.0011 (11)	−0.0098 (15)	0.0069 (13)
C6	0.0476 (15)	0.0196 (12)	0.031 (2)	0.0055 (11)	−0.0154 (16)	−0.0036 (11)
C7	0.0373 (14)	0.0263 (13)	0.027 (2)	0.0100 (10)	−0.0094 (14)	−0.0034 (12)
C8	0.0251 (10)	0.0237 (11)	0.0236 (16)	−0.0030 (9)	−0.0008 (14)	0.0022 (12)
C9	0.0282 (11)	0.0241 (12)	0.0247 (17)	−0.0007 (10)	0.0026 (13)	0.0017 (11)
C10	0.0261 (11)	0.0264 (12)	0.0291 (18)	−0.0005 (9)	−0.0006 (14)	−0.0011 (13)
C11	0.0240 (11)	0.0227 (12)	0.036 (2)	−0.0019 (9)	0.0002 (13)	0.0032 (11)
C12	0.0269 (12)	0.0258 (12)	0.033 (2)	0.0008 (10)	0.0037 (13)	0.0015 (12)
C13	0.0347 (13)	0.0286 (13)	0.041 (2)	−0.0020 (10)	0.0072 (16)	−0.0055 (14)
C14	0.0352 (14)	0.0214 (12)	0.058 (3)	0.0018 (10)	0.0138 (17)	−0.0036 (14)
C15	0.0345 (13)	0.0272 (13)	0.051 (2)	0.0053 (10)	0.0036 (16)	0.0082 (15)
C16	0.0339 (13)	0.0346 (15)	0.041 (2)	0.0037 (11)	−0.0025 (15)	0.0019 (13)

### Geometric parameters (Å, º)

**Table:**

S1—C1	1.662 (3)	C6—H6	0.9500
S2—C1	1.757 (3)	C7—H7	0.9500
S2—C2	1.815 (3)	C8—C9	1.444 (4)
N1—C1	1.339 (3)	C8—H8	0.9500
N1—N2	1.376 (3)	C9—C10	1.333 (4)
N1—H1N	0.880 (10)	C9—H9	0.9500
N2—C8	1.284 (4)	C10—C11	1.460 (3)
N3—C3	1.339 (4)	C10—H10	0.9500
N3—C4	1.340 (4)	C11—C16	1.392 (4)
N3—H3N	0.880 (10)	C11—C12	1.400 (4)
C2—C3	1.502 (4)	C12—C13	1.381 (4)
C2—H2A	0.9900	C12—H12	0.9500
C2—H2B	0.9900	C13—C14	1.396 (5)
C3—C7	1.384 (4)	C13—H13	0.9500
C4—C5	1.380 (4)	C14—C15	1.375 (5)
C4—H4	0.9500	C14—H14	0.9500
C5—C6	1.381 (4)	C15—C16	1.391 (4)
C5—H5	0.9500	C15—H15	0.9500
C6—C7	1.380 (4)	C16—H16	0.9500
			
C1—S2—C2	101.42 (13)	C6—C7—H7	120.2
C1—N1—N2	120.1 (3)	C3—C7—H7	120.2
C1—N1—H1N	123 (2)	N2—C8—C9	119.7 (3)
N2—N1—H1N	117 (2)	N2—C8—H8	120.2
C8—N2—N1	114.9 (3)	C9—C8—H8	120.2
C3—N3—C4	123.6 (2)	C10—C9—C8	123.4 (3)
C3—N3—H3N	122 (2)	C10—C9—H9	118.3
C4—N3—H3N	114 (2)	C8—C9—H9	118.3
N1—C1—S1	121.2 (2)	C9—C10—C11	127.1 (3)
N1—C1—S2	112.4 (2)	C9—C10—H10	116.4
S1—C1—S2	126.41 (16)	C11—C10—H10	116.4
C3—C2—S2	113.99 (18)	C16—C11—C12	118.1 (3)
C3—C2—H2A	108.8	C16—C11—C10	119.4 (3)
S2—C2—H2A	108.8	C12—C11—C10	122.6 (3)
C3—C2—H2B	108.8	C13—C12—C11	121.1 (3)
S2—C2—H2B	108.8	C13—C12—H12	119.5
H2A—C2—H2B	107.6	C11—C12—H12	119.5
N3—C3—C7	118.3 (3)	C12—C13—C14	119.8 (3)
N3—C3—C2	118.5 (2)	C12—C13—H13	120.1
C7—C3—C2	123.2 (3)	C14—C13—H13	120.1
N3—C4—C5	119.6 (3)	C15—C14—C13	119.9 (2)
N3—C4—H4	120.2	C15—C14—H14	120.1
C5—C4—H4	120.2	C13—C14—H14	120.1
C4—C5—C6	118.5 (3)	C14—C15—C16	120.1 (3)
C4—C5—H5	120.8	C14—C15—H15	119.9
C6—C5—H5	120.8	C16—C15—H15	119.9
C7—C6—C5	120.4 (3)	C15—C16—C11	121.0 (3)
C7—C6—H6	119.8	C15—C16—H16	119.5
C5—C6—H6	119.8	C11—C16—H16	119.5
C6—C7—C3	119.6 (3)		
			
C1—N1—N2—C8	−171.9 (2)	C2—C3—C7—C6	176.6 (3)
N2—N1—C1—S1	179.90 (19)	N1—N2—C8—C9	180.0 (2)
N2—N1—C1—S2	−1.0 (3)	N2—C8—C9—C10	179.4 (3)
C2—S2—C1—N1	176.37 (19)	C8—C9—C10—C11	179.2 (3)
C2—S2—C1—S1	−4.5 (2)	C9—C10—C11—C16	176.5 (3)
C1—S2—C2—C3	−76.9 (2)	C9—C10—C11—C12	−3.8 (5)
C4—N3—C3—C7	1.7 (4)	C16—C11—C12—C13	0.1 (4)
C4—N3—C3—C2	−176.4 (3)	C10—C11—C12—C13	−179.6 (3)
S2—C2—C3—N3	−52.0 (3)	C11—C12—C13—C14	0.8 (4)
S2—C2—C3—C7	129.9 (3)	C12—C13—C14—C15	−1.1 (5)
C3—N3—C4—C5	−1.1 (4)	C13—C14—C15—C16	0.5 (5)
N3—C4—C5—C6	0.1 (4)	C14—C15—C16—C11	0.5 (5)
C4—C5—C6—C7	0.1 (4)	C12—C11—C16—C15	−0.8 (4)
C5—C6—C7—C3	0.6 (4)	C10—C11—C16—C15	178.9 (3)
N3—C3—C7—C6	−1.5 (4)		

### Hydrogen-bond geometry (Å, º)

**Table:**

*D*—H···*A*	*D*—H	H···*A*	*D*···*A*	*D*—H···*A*
N1—H1*N*···Cl1^i^	0.88 (2)	2.29 (2)	3.104 (3)	153 (3)
N3—H3*N*···Cl1	0.88 (2)	2.13 (2)	2.9833 (19)	163 (3)

Symmetry code: (i) *x*, *y*, *z*−1.
