# Supplementary figures and images for: Crystal structure of 2-{[2-(3-phenyl­allyl­idene)hydrazin-1-yl]thio­carbonyl­sulfanylmeth­yl}pyridinium chloride
Source: Acta Crystallogr Sect E Struct Rep Online. 2014 Oct 29;70(Pt 11):o1207–8. doi: 10.1107/S1600536814023228 (PMC4257274; doi:10.1107/S1600536814023228)

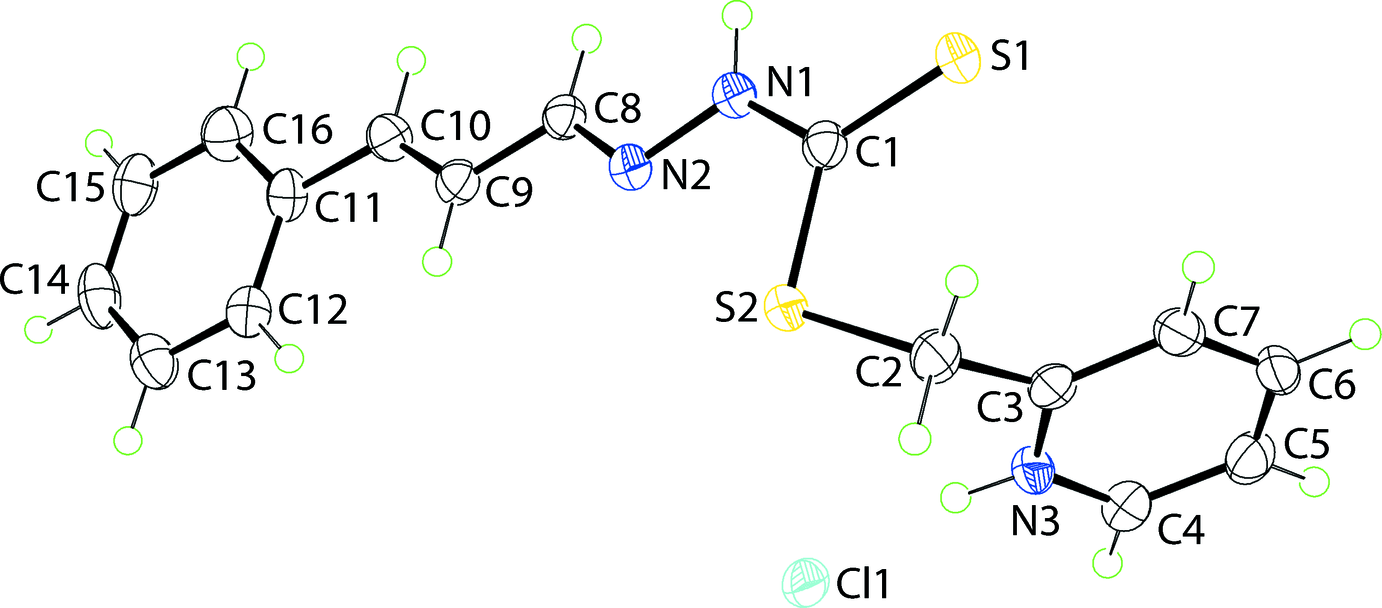

Supplement: Supplementary file 4 [file e-70-o1207-fig1.tif]

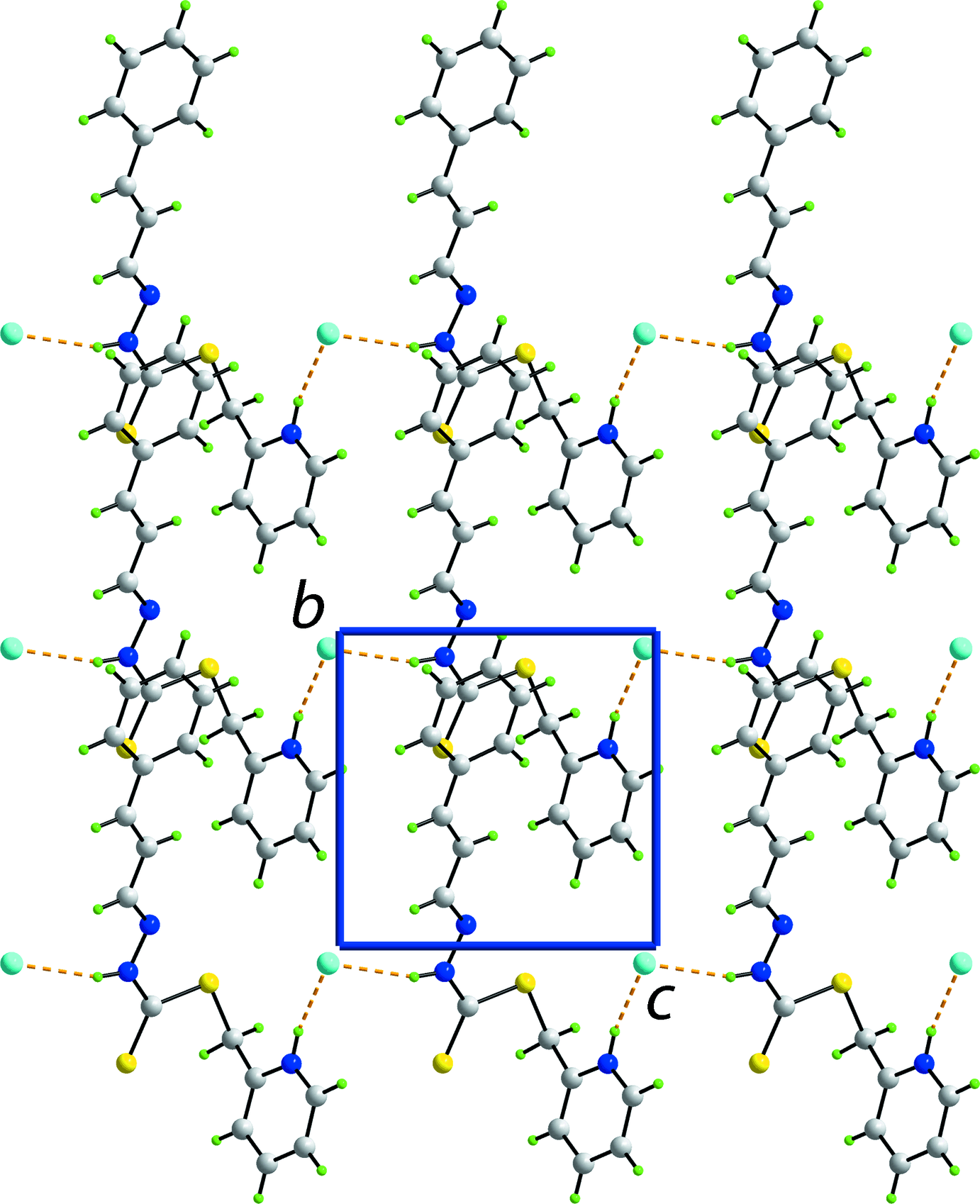

Supplement: Supplementary file 5 [file e-70-o1207-fig2.tif]
